# Black Seed Oil, Bentonite Clay, and Probiotics: A Comprehensive Holistic Cure for *Clostridium difficile* Infection in a 2-Year-Old Female Child

**DOI:** 10.1155/2022/2002488

**Published:** 2022-05-29

**Authors:** Emily Littman, Nicole Winningham, Tana B. Carson, Ivette M. Hidalgo

**Affiliations:** ^1^Occupational Therapy, University of Florida, Gainesville, FL 32611, USA; ^2^College of Medicine, University of Central Florida, Orlando, FL 32827, USA; ^3^Holistic Nutrition Services LLC, Austin, TX 78731, USA; ^4^Occupational Therapy, Florida International University, Miami, FL 33199, USA; ^5^Nursing, Florida International University, Miami, FL 33199, USA

## Abstract

There has been a rise in antibiotic resistance in secondary conditions such as *Clostridium difficile* (*C. difficile*) due to overuse of antibiotics. Oral antibiotics are used to treat *C. difficile*, which further disrupts the intestinal flora resulting in unwanted side effects. Naturopathic treatments often have fewer side effects and lower secondary infection risk than pharmaceutical interventions making them ideal for pediatric use. This case report describes the effective treatment of a pediatric clinical case of *C. difficile* using naturopathic and complementary alternative medicines (CAMs) including black seed oil (*Nigella sativa*), bentonite clay, and probiotics. A healthy two-year-old patient presented to a pediatrician with symptoms of, and subsequently confirmed, *C. difficile* after having been recently hospitalized and treated for a gluteal abscess and cellulitis using clindamycin, vancomycin, and piperacillin/tazobactam. Through a shared decision-making process, the patient's mother and providers developed a treatment plan for the *C. difficile* infection (CDI), which included black seed oil, bentonite clay, and *Lactobacillus* probiotics. No *C. difficile* was detected via stool immunoassay after 4 days of combined CAM therapy. Our results underscore the need for additional research regarding the effectiveness of naturopathic CAMs including black seed oil, bentonite clay, and probiotics as alternatives to antibiotic treatment of CDI in children.

## 1. Background

According to the Centers of Disease Control and Prevention, *Clostridium difficile* infection (CDI) has been categorized as an urgent threat due to increasing antibiotic resistance globally [1]. CDI has become one of the most common healthcare-associated infections, with approximately half a million people in the USA contracting *C. difficile* each year [2, 3]. Large longitudinal studies suggest costs for in-hospital management of CDI may range between $4.8–6.3 billion annually [4, 5].

*C. difficile*, a Gram-positive bacterium, creates toxins that attack the lining of the intestine, which may result in frequent and watery diarrhea, abdominal pain, and possible life-threatening conditions such as inflammation of the colon [6]. The most common cause of CDI is the use of antibiotics, however the incidence of CDI is also higher in individuals with a prior history of CDI, recent hospitalization, immunocompromisation, inflammatory bowel disease, or prolonged use of proton pump inhibitors [2, 7, 8].

Antibiotics, such as fluoroquinolones, cephalosporins, and can disrupt the intestinal flora, interrupting the equilibrium of over 100 trillion bacterial representing 2,000 different subtypes [2, 6]. This disruption of gut microflora creates an environment conducive to unregulated *C. difficile* growth, and the toxins produced result in damage to intestinal lining. This is particularly worrisome due to the emergence of new, antibiotic-resistant strains of *C. difficile* [2, 9, 10]. It has been suggested that conscientious antimicrobial prescribing along with identification of alternative methods to treat infections is essential in mitigating CDI development [11, 12].

Standard treatment guidelines for CDI call for the use of antibiotics; persons experiencing greater than 3 loose stools within 24 hours and a positive stool immunoassay for *C. difficile* require treatment [13–16]. Current guidelines suggest a 10-day regimen of oral vancomycin, metronidazole, or fidaxomicin [3, 14, 17, 18]. The need for antibiotics presents a health dilemma, as the new antibiotic may further disrupt intestinal flora and leaves the gut more susceptible to CDI recurrence weeks or even months following antibiotic cessation [2, 7, 19].

Prolific antibiotic use and antibiotic resistance has led to a rise in CDI prevalence, and cases in the pediatric population have been on the rise and their effects significant [2, 12, 20–22]. Children experiencing CDI may develop rapid leukocytosis, renal or hepatic impairment, and lactic acidosis in addition to gastrointestinal sequelae such as an ileus or toxic megacolon [14, 23–26]. As such, prompt recognition and treatment is essential to promote wellbeing in the pediatric population.

In light of the challenges associated with antibiotic use, patients are starting to turn towards more holistic treatments for their demonstrable antimicrobial action [7, 11]. CAM modalities should be evaluated for their potential as a suitable antibiotic alternative in cases of CDI. Some adult case studies have been reported regarding the use of various naturopathic remedies for CDI, however these are limited. There is a dearth of controlled trials evaluating a CAM protocol for CDI in children. Thus, little is known of the usefulness of CAM approaches to CDI treatment in the pediatric population [27]. We report a case of a child who developed CDI following antibiotic treatment for cellulitis, utilized a CAM regimen consisting of black seed oil, bentonite clay, and probiotics, and successfully overcame CDI without need for antibiotics.

## 2. Case Presentation

### 2.1. Patient

A previously healthy two-year-old female with no significant past medical or recent travel history presented to the pediatric Emergency Department (ED) accompanied by her parents with chief complaint of decreased level of activity, decreased oral intake, lethargy, and fever. Her mother reported development of a small “pimple” to the patient's right gluteal area three days prior to presentation, which had worsened in appearance and became painful on the day of arrival to the ED. The father, a paramedic, drained approximately 12 mL of purulent material from the wound prior to presentation in the ER. Vital signs obtained in the ED showed temperature of 37.8°C, patient was tachycardic at 104 bpm, tachypneic at 26 breaths/minute, hypotensive at 105/49 mmHg, and had an oxygen saturation of 97%. Physical exam revealed a large abscess with associated cellulitis. Labs were drawn, which were significant for leukocytosis (24.9 K/uL) and thrombocytosis (385 K/uL), and the patient was admitted. Pediatric surgery was consulted for incision and drainage of the gluteal abscess and wound cultures grew *Staphylococcus aureus*. The patient received intravenous clindamycin 120 mg, piperacillin/tazobactam 900 mg every 6 hours, and vancomycin 216 mg every 8 hours throughout her stay. The patient's condition improved after a three-day hospital stay, and she was discharged to home with prescribed oral clindamycin 120 mg three times daily and mupirocin 2% ointment to be applied to the wound three times daily for 7 days.

Five days after completion of antibiotic regimen, the patient began having frequent watery, mucus-containing stool. The patient and the family presented to the pediatrician two days later with concerns about these symptoms. Given the patient's history of recent antimicrobial treatment, particularly with clindamycin as the agent of choice, along with her presenting symptomatology, *C. difficile* infection was a leading differential diagnosis. Outpatient stool cultures were obtained, which were positive for *C. difficile*.

### 2.2. Treatment

The patient's pediatrician prescribed oral metronidazole suspension at 88.5 mg, four times a day for 14 days. The patient's mother was reluctant to initiate additional antibiotic therapy out of concern for worsening symptoms and chose to forgo antibiotic therapy and obtain additional opinions. The family consulted a holistic nutrition consultant holistic nutritionist, who reviewed available evidence-based CAM options for *C. difficile* treatment and diarrhea management. These options included binders, such as activated charcoal and bentonite clay, as well as black seed oil and probiotics. Together, the holistic nutrition consultant and parents identified black seed oil for its antibiotic effects on *C. difficile*, bentonite clay for symptomatic management of diarrhea, and specific strains of probiotics to restore intestinal flora [7, 28–30]. A 14-day regimen was recommended by the pediatrician. The family and pediatrician agreed to pursue the CAM treatment option; the patient's family was instructed by the pediatrician to monitor symptoms and begin prescribed metronidazole if symptoms persisted beyond three days or if the patient's condition worsened.

Dosages were estimated based on the patient's body weight (11.9 kg). Since there are no pediatric dosing recommendations for black seed oil, the patient's dose was calculated using adult body weight dosing. The patient's body weight was 1/6^th^ the average adult North American's body weight (80.7 kg); therefore, the patient was given approximately 1/8^th^ of the recommended adult dose of black seed oil as a conservative approach to estimating a pediatric dosage. [31] Probiotics including strains of *S. thermophilus, L. bulgaricus, L. acidophilus, Bifidus, L. paracasei and L. rhamnosus* were dosed based on nutrition facts listed on the label of the individual probiotic product in yogurt form (Table 1). A chewable tablet containing *Lactobacillus GG* was also recommended. Evidence-based guidelines exist for the dosing of bentonite clay in cases of infectious diarrhea, however evidence is scarce related to its use for CDI. As such, both evidence and labeling instructions were considered to determine appropriate dosing in this case.

## 3. Results

### 3.1. Follow-up

Per caregiver report, the patient's symptoms subsided within 3 days. The patient's diarrhea improved within 1 day and resolved by day 3. The patient's mother also reported that the CAM treatment was well tolerated, and the patient did not experience any unwanted side effects associated with the naturopathic treatment.

### 3.2. Post-Treatment Stool Immunoassay

A stool immunoassay for *C. difficile* toxin/glutamate dehydrogenase (GDH) with reflex to polymerase chain reaction (PCR) was completed four days after starting naturopathic treatment. The stool immunoassay did not detect *C. difficile* toxin A and B nor *C. difficile* antigens. Although symptoms resolved after 3 days of treatment, the patient continued the CAM protocol for 14 days, which is comparable to the timeline for standard care with antibiotics for CDI. At a three month follow up with the pediatrician, the patient showed no recurrence of symptoms.

## 4. Discussion

We present a case of a two-year-old patient that developed symptomatic CDI following a gluteal abscess and cellulitis that was treated with clindamycin, vancomycin and piperacillin/tazobactam through the course of hospitalization and discharged with an additional regimen of clindamycin. The patient contracted CDI following treatment for the cellulitis. Wanting to avoid further antibiotic therapy and mitigate potential for side effects, the patient's parents opted to consult with a holistic nutrition consultant and utilize CAM modalities, which promptly eradicated the CDI without further incident.

CDI is a possible complication following antibiotic therapy for a myriad of infections. Clindamycin, in particular, has been shown to increase the risk of CDI development by 16-fold [32]. The wide range of possible short and long-term side effects associated with treating CDI with typical antimicrobial regimens and the emergence of antibiotic-resistant strains highlights the importance of CAM therapy as a potentially valuable treatment option.

*Nigella sativa* (black seed oil) is an herbaceous plant that has been used for thousands of years in traditional medicine as a protective and curative remedy [33, 34]. Over 100 compounds make up black seed oil including volatile oils, fatty acids, flavonoids, saponins, and proteins [35, 36]. The primary active ingredient, thymoquinone, specifically combats anaerobes such as *C. difficile* in addition to its antimicrobial activity against other Gram-positive and Gram-negative aerobes [28, 35]. Its use is further advantageous because levels do not appear to be affected by acidity levels found in the stomach [28]. Our data supports those generated by in vitro studies conducted by Randhawa et al., which showed the efficacy of black seed oil against *C. difficile* [37].

Bentonite clay, a result of volcanic ash, in addition to other clays have been used both internally and externally in traditional medicine for centuries to treat diseases [29]. Clay has an antimicrobial effect on drug-resistant pathogens with no toxic side effects [38]. Bentonite clay specifically binds to and neutralizes *C. difficile* protein toxins and has been used to treat diarrhea and cholera [39, 40]. Numerous studies have shown the efficacy of bentonite clay at ameliorating when used as a standalone option [40–42]. Smectite, a form of bentonite clay, has been shown to reduce duration of diarrhea by one day and lead to full resolution by day three in pediatric patients [42]. Our results support the work of Perez-Gaxiola et al., which showed the use of clay for treatment of diarrhea to be effective [42].

Probiotics, which are live bacteria, such as lactobacilli can also be used to treat *C. difficile* by restoring balance to the intestinal flora [11, 43]. Probiotics also help limit the growth and toxicity of harmful bacteria like *C. difficile* by competing for nutrients [11]. Researchers suggest that when a patient takes probiotics during an antibiotic regimen, the risk of *C. difficile* associated diarrhea decreases by approximately 60% [44]. A case study using an animal model provided evidence that the probiotic *Lactobacillus acidophilus* produces lactic acid which decreases pH in the gut and suppresses *C. difficile* growth [45]. Our results suggest the efficacy of probiotics when used in conjunction with other CAM modalities. Evidence is mounting in support of probiotic use to prevent opportunistic bacterial infections, however the extent to which probiotics assist in the specific, standalone treatment of CDI warrants further investigation [44].

### 4.1. Implications for Clinical Practice

The use of CAM is valuable because it reduces the frequency and duration of prescription medication use in addition to unwanted side effects [46–48]. CAM-containing natural products with antibacterial compounds can inhibit the pathogens from becoming resistant [19, 28]. Some natural products have been reported to be safer, more cost-effective, and more readily available to consumers. [7] For these reasons, the use of CAMs in the pediatric population is on the rise [27].

Despite its increase in popularity, numerous barriers to the implementation of CAM therapies exist. Common barriers include limited access to information about CAM due to poor patient-provider communication, limited research regarding efficacy and dosing, specifically in the pediatric population, and/or a lack of provider education or training. One study found 67.5% of parents of pediatric patients reported using CAM therapies but endorsed a lack of education or communication from a healthcare professional prior to its initiation. [49] According to Huang, parents reported physician's perceived knowledge in CAMs and interest in learning more about the CAMs was low when caring for their child [50]. In a study of pediatric residents, 88% of respondents perceived a gap in knowledge pertaining to CAM modalities due to lack of educational exposure [51].

As a result, patients and families are left to explore CAM options independently or forced to form and coordinate a team of professionals who can instruct about its use. For pediatric providers with a lack of knowledge, referrals to a skilled professional, such as a certified holistic nutrition consultant, may be necessary to safely guide patients in exploring CAM options. Such interprofessional collaboration may further CAM research and expand the overall CAM knowledge base.

It is understood that up to 50% of infants can be asymptomatic carriers of *C. difficile*, with many children eradicating the bacteria by age two, secondary to a more robust, adult-like gut microbiome [52, 53]. Symptomatic CDI, however, is becoming more prevalent in healthy pediatric populations, leading parents, and caregivers to seek alternatives to antibiotics more frequently.

This case report is valuable for the insight it provides regarding successful CAM utilization in pediatric CDI. The provider's decision to limit further workup to definitively rule out other causes of the child's diarrhea, however, is a known limitation of this case report. Additionally, further clinical research regarding CAM effectiveness and dosage is needed to increase the knowledge base of CAM therapies and promote safe exploration and utilization of these options.

## 5. Conclusions

The clinical case of CDI reported here is an example of the effective use of CAM for treating an infectious condition without the unwanted side effects associated with antibiotics. Additionally, this case highlights the clinical value of the parent-provider relationship as well as the unique challenges that both parents and providers must overcome to successfully implement CAM in pediatrics. In this case, the caregivers of a pediatric patient with CDI combined information from several sources to develop a successful pediatric CAM regimen in order to make choices regarding a plan of care for the child. Although this protocol was disclosed to, and approved by, the patient's primary care provider and holistic nutrition consultant, it was developed by the patient's family. This approach was both appropriate and effective in this case. In other cases, however, it may not be feasible for families to coordinate care in this way. Therefore, if alternative options are requested by patients or their families, pediatricians should be prepared to either guide families through appropriate CAM options or to consult with other professionals while continuing to coordinate and monitor the patient's care. This case demonstrates that it may be reasonable, when patients are otherwise healthy, to trial black seed oil in combination with probiotics and bentonite clay to treat CDI. Further research is warranted regarding the use of black seed oil, bentonite clay, and probiotics for *C. difficile* treatment to determine effectiveness and establish proper dosages for this type of treatment across the lifespan.

## Figures and Tables

**Table 1:** Treatment regimen for two-year-old patient.

Treatment regimen	
Morning *(upon waking)*	
1.	1/8^th^ tsp of fermented black seed booster powder (new chapter®)
	½ teaspoon of 500 mg organic N. sativa (black seed), mixed with fruit juice to drink
2.	(1) stonyfield organic yogurt cup with probiotics
3.	(1) tablet Culturelle® kids chewable tablets
Afternoon *(upon waking from nap)*	
1.	0.7 mL of black cumin seed oil (N. Sativa health from the sun®)
	Mixed with juice and honey to drink.
2.	½ capsule of saccharomyces boulgaris + MannanOligoSaccharides (MOS).
3.	*Saccharomyces* boulardii (a *S. cerevisiae* strain, 5 billion viable cells per capsule)
4.	Bio-MOS (200 mg per capsule, derived from *S. cerevisiae*, source of MOS; jarrow formulas®)
Evening *(before sleep)*	
1.	½ capsule of saccharomyces boulgaris + MannanOligoSaccharides (MOS). *Saccharomyces* boulardii (a *S. cerevisiae* strain 5 billion viable cells per capsule)
	Bio-MOS (200 mg per capsule, derived from *S. cerevisiae*, source of MOS; jarrow formulas®)
2.	½ tablet of *Lactobacillus Acidophilus La-14* (100 million cfu per tablet) 0.5 mg tablet (Nature's Bounty®)
3.	(1) tablet culturelle ® kids chewable tablets
4.	¼ tsp bentonite clay liquid (ingredients per tablespoon: Iron 2 mg, sodium 10 mg, purified bentonite GP premium grade USP/NF hydrated with filtered water 15 ml, yerba Prima®) mixed with ¼ tsp honey and 5 mL water to drink

## Data Availability

Due to the nature of this research, participants of this study did not agree for their data to be shared publicly other than what is included in this report, so supporting data is not available.
